# Short Term Treatment Monitoring of Renal and Inflammatory Biomarkers with Naturally Occurring Leishmaniosis: A Cohort Study of 30 Dogs

**DOI:** 10.3390/vetsci11110517

**Published:** 2024-10-22

**Authors:** Valeria Pantaleo, Tommaso Furlanello, Michela Campigli, Laura Ventura, Laia Solano-Gallego

**Affiliations:** 1 San Marco Veterinary Clinic and Laboratory, Via dell’Industria 3, 35030 Veggiano, Italy; valeriapant@libero.it (V.P.); tf@sanmarcovet.it (T.F.); michelacampigli@virgilio.it (M.C.); 2 Departament de Medicina i Cirurgia Animals, Universitat Autònoma de Barcelona, 08193 Cerdanyola de Vallès, Spain; 3 Department of Statistical Science, University of Padua, Via Cesare Battisti 241, 35121 Padua, Italy; ventura@stat.unipd.it

**Keywords:** antithrombin, C-reactive protein, *Leishmania infantum*, severity, total iron-binding capacity, urinary amylase-to-creatinine ratio

## Abstract

Various inflammatory and renal biomarkers have already been assessed for monitoring the response to anti-leishmanial therapy in canine leishmaniosis. This study assessed the parasite load, various inflammatory and renal biomarkers pre- and post-treatment, and any association between the studied variables and the degree of disease severity at diagnosis. This is a prospective cohort study of 30 client-owned dogs with leishmaniosis, classified according to LeishVet’s guidelines as stage I (n = 2), stage IIa (n = 7), stage IIb (n = 6), stage III (n = 8), and stage IV (n = 7). In addition to *Leishmania* real-time PCR in the bone marrow, blood and urine, previously studied biomarkers, and several inflammatory and renal markers never investigated in canine leishmaniosis, such as fibrinogen, antithrombin, urinary fractional excretion of sodium, and urinary amylase-to-creatinine ratio were measured pre- and post-treatment (meglumine antimoniate or miltefosine + allopurinol). A positive *Leishmania* real-time PCR in the blood at diagnosis predicted a positive *Leishmania* real-time PCR in the bone marrow post-treatment (*p* = 0.003). Following treatment, antithrombin and urinary amylase-to-creatinine ratio were significantly changed (*p* < 0.001, respectively). Urinary amylase-to-creatinine ratio, total iron-binding capacity, and antithrombin were the variables most strongly associated with disease severity (*p* < 0.005, respectively). Urinary amylase-to-creatinine ratio can be a useful marker to monitor treatment response and to classify the degree of disease severity.

## Simple Summary

Canine leishmaniosis can cause inflammation and renal disease that is considered the main cause of death in dogs with leishmaniosis. In the past, various biomarkers have been investigated to assess the severity of the disease and the response to anti-*Leishmania* treatment; however, the need for new markers remains. In the study, 30 dogs diagnosed with leishmaniosis were divided into different groups based on the degree of the disease and evaluated at diagnosis and after anti-*Leishmania* treatment. Parasite load in the bone marrow, blood, and urine, previously investigated, as well as new inflammatory and renal biomarkers were evaluated before and post-treatment. Treated dogs showed a significant decrease in the parasite load in the various tissues evaluated and a significant variation of various inflammatory and renal biomarkers. Among various biomarkers, a new one was identified and could be useful to monitor treatment response and to classify disease severity at the time of diagnosis.

## 1. Introduction

Canine leishmaniosis (CanL) is a protozoan disease caused by *Leishmania infantum* (*L. infantum*), exhibiting highly variable degrees of severity [1,2]. Renal disease can be the sole manifestation of the disease, and it is considered the most common cause of death in CanL [3,4,5]. Inflammation and renal injury are expected with disease progression [6,7]. The efficacy of treatment is not always easy to assess, and for the most part, it is based on the improvement of clinical signs and clinicopathologic abnormalities since a parasitological cure is rarely achieved [8,9,10]. Numerous inflammatory and renal biomarkers have been previously evaluated for monitoring the response to anti-leishmanial treatment, such as C-reactive protein (CRP), serum amyloid A, haptoglobin (Hp), and proteinuria [11].

Acute phase proteins (APPs) are plasma proteins whose concentrations in blood change in response to inflammation, providing valuable information in the monitoring of various diseases [12,13]. A decrease in positive APPs, including CRP, Hp, and serum amyloid A was reported following anti-leishmanial treatment in both experimentally and naturally infected dogs [14,15]. In the experimental infection of dogs with *L. infantum*, an increase in ferritin (Ft) and a decrease in paraoxonase-1 (PON-1) was observed with a return to pre-infection values following treatment [16,17]. In one study, changes in serum albumin (Alb) and other APPs were evaluated in dogs with leishmaniosis after receiving either antimonials and allopurinol or miltefosine and allopurinol [18].

Other studies have also explored changes after anti-leishmanial treatment in serum and urinary renal biomarkers, such as urea and creatinine (Cr), symmetrical-dimethylarginine (SDMA), urine protein-to-creatinine ratio (UPC), and other markers [9,19,20,21,22,23]. Creatinine, UPC, urine specific gravity (USG), microalbuminuria, urinary concentration of cystatin C, and lipocalin-2 were evaluated during CanL treatment with miltefosine alone or together with allopurinol [21]. Furthermore, the *Leishmania* parasitic load assessed by real-time polymerase chain reaction (q-PCR) in different tissues has been used to demonstrate the infection and also to evaluate the response to anti-*Leishmania* therapy [22,24]. Existing biomarkers have limitations, and they may not be ideal for all stages of leishmaniosis, emphasizing the importance of new inflammatory and renal biomarkers for more effective monitoring of treatment response.

The aims of this study were to assess the following parameters before and after treatment: (1) the parasitic load in the bone marrow, blood, and urine; (2) several inflammatory markers never investigated before in CanL and including fibrinogen (Fg) and antithrombin (AT) alongside others previously reported [14,15,16,17,18,19,25]; (3) two renal biomarkers never investigated before in CanL and including urinary fractional excretion of sodium (FeNa) and the urinary amylase-to-creatinine ratio (uAm/Cr) alongside others previously reported [9,21,22,23] and to evaluate any possible associations between the studied variables and the degree of disease severity at the time of diagnosis according to the LeishVet clinical staging [3,26,27].

## 2. Materials and Methods

### 2.1. Dogs and Study Design

This was a prospective cohort study of client-owned dogs diagnosed with *L. infantum* infection after admission to the San Marco Veterinary Clinic between April 2020 and December 2021.

CanL was diagnosed based on presence of compatible clinical and laboratory abnormalities [3,26], positivity for *L. infantum*-specific antibodies using a commercial enzyme-linked-immunosorbent-assay (ELISA) (VetLine *Leishmania*, *Leishmania* ELISA test, NovaTec Immunodiagnostica GmbH, Dietzenbach, Germany) [28] and a positive *Leishmania* q-PCR on bone marrow [29]. Criteria for inclusion into the study were: (1) a diagnosis of CanL and absence of previous treatment with conventional anti-*Leishmania* drugs or immunomodulators; (2) availability of a complete blood count (CBC), serum biochemistry, coagulation profile, and urinalysis; (3) negative antigenic test for *Dirofilaria immitis* (Filarcheck 96, biopronix by Agrolabo, Italy) as well as negative antibodies against *Anaplasma phagocytophilum*, *Ehrlichia canis*, and *Rickettsia conorii* antigens (semiquantitative immunofluorescence by MegaFLUO ANAPLASMA ph. MEGACOR; MegaFLUO EHRLICHIA canis MEGACOR; MegaFLUO RICKETTSIA conorii MEGACOR; Hörbranz, Austria); (4) inactive urine sediment; (5) exclusion of other concurrent diseases based on clinical examination and blood and urinalysis results; (6) no administration of any type of drug in the previous three months; and (7) no previous administration of CanL vaccine.

At the time of diagnosis, all the dogs were classified according to the Leishvet guidelines [3] and the International Renal Interest Society (IRIS) recommendations for chronic kidney disease [30]. Subsequently, all the dogs were divided into two groups: dogs with mild to moderate disease included dogs classified in LeishVet stage I, IIa, or IIb (Group 1, n = 15) and dogs with severe to very severe disease dogs classified in LeishVet stage III or IV (Group 2, n = 15). All clinical signs documented at diagnosis were monitored over time. The following plan was scheduled with each owner: a telephone update at day 15, a clinical visit at day 30 (at the end of the leishmanicidal therapy), and a telephone update at day 120.

### 2.2. Treatment Protocol and Follow-Up

Based on the clinician’s decision and the owner’s willingness to comply with treatment, some dogs (n = 23) received 50 mg/kg of meglumine antimoniate [(MA), Glucantime, Boehringer Ingelheim, Germany] subcutaneously every 12 h for 28 days and 10 mg/kg of allopurinol [(A), Allopurinol DOC, Italy] orally twice daily for 6 months, and other dogs (n = 7) received 2 mg/kg of miltefosine [(MT), Milteforan, VIRBAC, Spain] orally every 24 h for 28 days and 10 mg/kg of A orally twice daily for 6 months [3]. All the diagnostic tests were carried out at diagnosis and at day 30 if dogs were treated with MA + A [8] or at day 60 if treated with MT + A [31]. Dogs with severe to very severe disease were subdivided based on the treatment protocol in dogs treated with MA + A (Group 3, n = 9) and dogs treated with MT + A (Group 4, n = 6).

### 2.3. Sample Collection and Diagnostic Tests

A blood sample was collected via cephalic or jugular venipuncture into 10 mL sterile plastic syringes, and 2 ml of blood was transferred to plastic tubes containing K_3_-EDTA for a CBC carried out in an automated hematology analyzer (ADVIA 2120i, Siemens, Erlangen, Germany). Then, 5 ml of blood was placed into serum glass tubes for chemical analysis carried out using an automated biochemical analyzer (Atellica^®^ Solution, Siemens, Germany) and 2 mL of blood into plastic tubes containing 3.2% sodium citrate for a coagulation profile carried out using an automated coagulation analyzer (BCSXP, Siemens, Germany). Next, 10 ml of urine was obtained by free-catch for urinalysis, and urinary chemistry was carried out using an automated biochemical analyzer (Atellica^®^ Solution, Siemens, Germany). After urine centrifugation, the USG was determined with a refractometer (reference interval > 1030). The urinary sediment was analyzed using an optical microscope to exclude urine samples with active sediment (>5 white blood cells per high power field [hpf], >5 red blood cells/hpf or any visible bacteria). The UPC was determined by calculating the ratio between the microproteins and the Cr measured in the urinary supernatant. Urine proteins (UPs) were measured in an automated spectrophotometer (Atellica^®^ Solution, Siemens, Germany) using pyrogallol red (Atellica CH Urinary/Cerebrospinal Fluid Protein (UCFP), Siemens Healthcare Diagnostics Inc., Tarrytown, NY, USA) and uCr with a modified Jaffe method (Siemens Healthcare Diagnostics Inc., USA). Symmetric-dimethylarginine was measured using an SDMA ELISA test (Eurolyser Diagnostica GmbH, Salzburg, Austria). The variables white blood cell count (WBC), PON-1, Hp, Ft, CRP, iron, TIBC, Alb, globulins (Glob), Fg, AT, urea, Cr, SDMA, USG, UPC, FeNa, uAm/Cr, urinary glucose-to-creatinine ratio (uG/Cr), urinary γ-glutamyl-transferase-to-creatinine ratio (uGGT/Cr), urinary ferritin-to-creatinine ratio (uFerr/Cr), *Leishmania* q-PCR in bone marrow aspirates, whole blood, and whole urine were measured at diagnosis and at day 30 or 60 depending on the treatment chosen based on the clinician’s decision and the owner’s willingness to comply with treatment.

A *Leishmania* ELISA test was carried out for the detection of *L. infantum* antibodies following the manufacturer’s instructions [32]. A dog was defined as seropositive if the antibody levels were >11%.

Bone marrow aspirates were obtained from the costochondral junctions by two experienced operators working at the clinic using an 18-gauge needle connected to a 10 mL syringe according to the protocol described by Paparcone and colleagues for the diagnosis and monitoring of CanL [33]. The bone marrow aspiration was performed in each dog positioned on lateral recumbency or standing (depending on the position preferred by the dog) in the presence of the owner, without any sedation, and lasted less than 2 min. The medullary juice of each dog was evaluated under a microscope by a clinical pathologist to confirm the adequacy of the sample before proceeding with a PCR assay for the detection of leishmanial DNA.

The extraction of the DNA was carried out using a High Pure PCR Template Preparation Kit (Roche Science Applied), according to the manufacturer’s protocol. Real-time PCR was carried out using LightCycler FastStart DNA Master^PLUS^ Hybridization Probes (Roche, Mannheim, Germany), utilizing a LightCycler version 3.5.17 instrument (Roche, Mannheim, Germany). Commercial *L. infantum* primers and a hybridization Taqman MGB probe, which amplified a fragment of the kinetoplast minicircle, were used. The thermal profile was 50 °C for 2 min and 95 °C for 10 min, followed by 40 cycles of 95 °C for 15 s and 60 °C for 60 s. Positive and negative controls were used in all the q-PCR runs as previously reported [29]. The results were positive if >100 copies of kinetoplast/mL were detected. The limit of detection was established according to the definition proposed by the Clinical Laboratory Standards Institute [34].

### 2.4. Sample Size

A priori power calculation was performed for a primary outcome for a Mann–Whitney test, with effect size P(X > Y) = AUC = 0.8, significance level 0.05, one-sided alternative hypothesis, and 12 subjects for each group provides a power of 0.8. No adjustments for multiple comparisons were a priori considered since, in this study, the number of possible variables useful to discriminate between the two groups was not known. However, considering two variables and the Bonferroni correction, we obtained a power of 0.8 with 15 subjects for each group.

### 2.5. Statistical Methods

The qualitative data were summarized using percentages and the quantitative using means and standard deviations (SDs), or median and interquartile range (IQR), according to the distributional assumptions. The normality of the distribution of the quantitative variables was assessed using the Shapiro–Wilk test. Variations of quantitative variables were assessed using the Student’s t-test or the Wilcoxon test for paired data, according to the distributional assumptions. With normality, the Student’s t-test was used to assess the significant differences between the groups of subjects, while without normality, the Mann–Whitney rank test was used. Differences in medians/means and 95% confidence intervals were calculated as effect size both for paired and independent comparisons, in the latter case using Group 1 as the reference group. Frequencies of qualitative variables were calculated utilizing McNemar’s test for dependent proportions. Associations between the qualitative variables were assessed using Pearson’s chi-squared test. The relationship between the quantitative variables was measured using Spearman’s correlation index. A receiver operating characteristic (ROC) curve and the area under the receiver operator characteristic curve (AUC-ROC) were used to assess the ability to differentiate the dogs in Group 1 from the dogs in Group 2 for the variables, which proved to be statistically different between the groups at the time of diagnosis. The accuracy of the test was classified as excellent (AUC > 0.9), good (0.8 < AUC ≤ 0.9), fair (0.7 < AUC ≤ 0.8), poor (0.6 < AUC ≤ 0.7), or failed (0.5 < AUC ≤ 0.6). The Youden procedure was applied to determine the best threshold value. A forward regression was used to model the two categories of subject groups (Groups 1 and 2) according to the various variables. The AUC was used to validate the fitted logistic regression model, and the goodness-of-fit was assessed by the Hosmer and Lemeshow test. Finally, a logistic regression model was also used to predict a positive *Leishmania* q-PCR in the bone marrow after leishmanicidal therapy, starting from a positive *Leishmania* q-PCR in the blood and urine at diagnosis. Statistical significance was set at *p* < 0.05. The data were analyzed using statistical R software, version 4.3.2.

## 3. Results

### 3.1. Clinical, Serological, and Molecular Data as Well as Individual Data at the Time of Diagnosis and Post-Treatment

Thirty dogs met the inclusion criteria. In total, 18 dogs were males (13 intact and 5 neutered), and 12 were females (5 intact and 7 spayed). The median age was 67.8 months (range 16.8–136.8 months), and the median body weight was 19.4 kg (range 5.7–42 kg). There were 13 mixed-breed dogs and 17 pure breed dogs (4 German shepherds, 2 dachshunds, 2 Labrador retrievers, 2 greyhounds, 1 Rottweiler, 1 border collie, 1 Kurzhaar, 1 English setter, 1 Akita Inu, 1 Siberian husky, and 1 beagle).

Dogs were classified based on the LeishVet and IRIS staging systems [3,26,27,30]. Serology, the parasitic load measured by q-PCR in bone marrow, blood, and urine and the treatment protocol implemented in each dog are reported in Supplementary Materials, Tables S1 and S2.

Two dogs were classified in LeishVet stage I, seven in stage IIa, six in stage IIb, eight in stage III, and seven in stage IV. Subsequently, all the dogs were divided into Group 1 (n = 15) and Group 2 (n = 15). In addition, 22 dogs were classified in IRIS stage I (10 dogs proteinuric and 9 hypertensive), 5 in stage III (4 proteinuric and 3 hypertensive), 1 in stage III (proteinuric and non-hypertensive), and 2 in stage IV (both proteinuric and hypertensive).

The *Leishmania* q-PCR in the bone marrow, blood, and urine was positive at the time of diagnosis in 30 (100%), 19 (63%), and 9 (30%) dogs, respectively. The *Leishmania* q-PCR in the blood was positive in 3/7 (43%) dogs in Leishvet stage IIa, 4/6 (67%) dogs in stage IIb, 6/8 (75%) dogs in clinical stage III, and 6/7 (86%) dogs in clinical stage IV. The *Leishmania* q-PCR on the urine was positive in 5/8 (62.5%) dogs in Leishvet stage III and 4/7 (57%) dogs in Leishvet stage IV (Supplementary Materials, Table S1). The frequency of the positive *Leishmania* q-PCR was significantly lower in the post-treatment samples as compared to the pre-treatment samples with 17/30 (57%), 2/19 (10.5%), and 2/9 (22%) dogs still positive in the bone marrow, blood, and urine, respectively (*p* < 0.001; *p* < 0.001; *p* = 0.04, Supplementary Materials, Table S2).

A logistic regression analysis selected a positive *Leishmania* q-PCR in the blood at the time of diagnosis (OR = 16.8, 95% CI = 2.9–146.6, *p* = 0.003) as a predictor for a positive *Leishmania* q-PCR in the bone marrow after leishmanicidal therapy. The fitted model provided an accuracy of 0.8, sensitivity of 0.88, and specificity of 0.7.

The most common clinical signs recorded in the dogs at the time of diagnosis and then monitored until the completion of therapy were skin lesions, such as papular dermatitis, onychogryphosis, periocular alopecia, exfoliative dermatitis and ulcerative dermatitis (n = 10), reduced appetite (n = 7, 3 of which also had polydipsia and polyuria), weight loss (n = 5), lameness (n = 3), lethargy (n = 2), blepharitis (n = 1), epistaxis (n = 1), and lymphadenomegaly (n = 1). At the end of the anti-leishmanial treatment, all the dogs had clinical remission except for the three dogs (dog ID 25, 29 and 30, see Supplementary Materials, Table S2) initially presented with reduced appetite, polydipsia, and polyuria, which were still polydipsic and polyuric. At the time of diagnosis, these three dogs were classified as Leishvet stage IV, and according to IRIS staging, one dog was classified as IRIS stage II and two dogs as IRIS stage IV. At presentation, they had a positive *Leishmania* q-PCR in the bone marrow, blood, and urine. After anti-leishmanial treatment, these three dogs were still in the same Leishvet and IRIS stages and were *Leishmania* positive in the bone marrow. One dog was also *Leishmania* positive in the blood and urine post-treatment. After 120 days, on telephone follow-up, the dogs’ owners confirmed that all dogs were still alive and had a good quality of life with supportive therapy provided in accordance with the IRIS guidelines [30].

### 3.2. Relationship Between Molecular, Inflammatory, and Renal Markers at the Time Diagnosis and Post-Treatment

**Figure 1:**
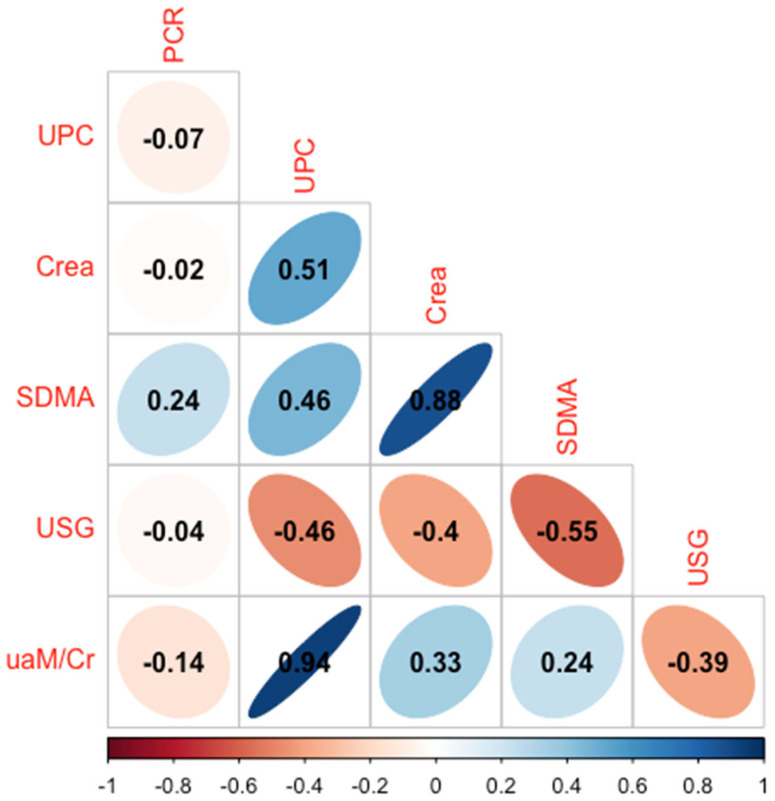
Correlation plot between urine *Leishmania* q-PCR and various renal biomarkers at diagnosis. PCR, urine *Leishmania*-PCR; UPC, urine protein-to-creatinine ratio; SDMA, symmetrical-dimethylarginine; USG, urine specific gravity; uAm/Cr, urinary amylase-to-creatinine ratio.

At diagnosis, there was a moderate correlation between the *Leishmania* q-PCR in the urine and Cr and SDMA (r = 0.58; *p* = 0.02; r = 0.67; *p* = 0.007) but not with the renal markers urea, UPC, USG, and uAm/Cr. There was also a very strong correlation (r = 0.94; *p* < 0.001) between the uAm/Cr and UPC and a strong correlation (r = 0.88; *p* = 0.003) between Cr and SDMA (Figure 1).

At diagnosis, no significant correlation was appreciated between age and the renal markers urea (*r* = −0.01, *p* = 0.95), Cr (*r* = 0.04, *p* = 0.76), SDMA (*r* = 0.03, *p* = 0.83), USG (*r* = −0.13, *p* = 0.32), and UPC (*r* = −0.12, *p* = 0.39).

In the first part of the study, the overall canine population was evaluated, and the WBC, TIBC, PON-1, and AT were significantly increased post-treatment as compared to pre-treatment (*p* = 0.01; *p* = 0.03; *p* = 0.02; *p* < 0.002, respectively, Table 1). The *Leishmania* q-PCR load in all the samples tested, CRP, Ft, USG, UPC, uAm/Cr, and uGGT/Cr were all significantly decreased post-treatment as compared to their pre-treatment values (*p* < 0.001; *p* < 0.001; *p* = 0.01; *p* = 0.02; *p* < 0.001; *p* = 0.008; *p* = 0.02; *p* < 0.001; *p* = 0.02, respectively, Table 1).

### 3.3. Evaluation of Molecular, Inflammatory and Renal Markers Based on Disease Severity

Subsequently, when dogs were classified as Groups 1 and 2, the same variables were evaluated again in both groups. The WBC, PON-1, and TIBC were significantly increased (*p* = 0.02; *p* = 0.002; *p* = 0.01, respectively, Table 2), and *Leishmania* parasitic load in the urine, CRP, USG, and UPC significantly decreased only in Group 2 post-treatment (*p* = 0.009; *p* = 0.004; *p* = 0.008; *p* = 0.02, respectively, Table 2, Table 3 and Table 4). At the end of the treatment, AT increased significantly in Group 1 and Group 2 (*p* = 0.007; *p* = 0.03, respectively, Table 2), while a significant decrease in the *Leishmania* parasitic load in the bone marrow (*p* < 0.001; *p* < 0.001, respectively, Table 3), blood (*p* = 0.02; *p* = 0.003, respectively, Table 3), Ft (*p* = 0.008; *p* = 0.006, respectively, Table 2), and uAm/Cr (*p* = 0.02; *p* = 0.003, respectively, Table 4) was observed in both groups.

The results of molecular, inflammatory, and renal markers of dogs with severe very severe disease divided by the treatment instituted (Group 3, MA + A, n = 9; and Group 4, MT + A, n = 6) are displayed in Supplementary Materials, Table S3. In both groups, there was a decrease in bone marrow and blood *Leishmania* q-PCR and Ft at the end of the treatment (*p* < 0.04, respectively). The WBC, PON-1, and TIBC increased significantly in dogs treated with MA + A (Group 3) (*p* < 0.02, respectively). In dogs in Group 3, Glob, USG, UPC, and uAm/Cr were significantly decreased (*p* < 0.02, respectively), while CRP and Cr were decreased only in dogs in Group 4 (MT + A) (*p* < 0.04, respectively).

Of all the variables studied, at diagnosis, the uAm/Cr, TIBC, AT, *Leishmania* q-PCR in the urine and CRP were the best parameters for detecting the severity of the disease with statistical significance. Based on the ROC curves, the uAm/Cr, TIBC, AT, *Leishmania* q-PCR in the urine, and CRP showed good accuracy (Table 5), while Alb, urea, SDMA, PON-1, uFerr/Cr, *Leishmania* q-PCR in the blood, and Fb showed fair accuracy in differentiating between Group 1 and Group 2 (Table 5).

A stepwise-forward logistic regression analysis selected uAm/Cr (OR = 1.1, 95% CI, 1.01–1.2, *p* = 0.03) and TIBC (OR = 0.99, 95% CI, 0.98–0.99, *p* = 0.01) as predictors of the group. The fitted model provided an AUC of 0.90, and the *p*-value of the Hosmer and Lemeshow goodness of fit was 0.836. The chance of developing the disease increases by 10% for each one-unit increase in uAm/Cr and decreases by 1% for each one-unit decrease in TIBC.

## 4. Discussion

To the extent of the authors’ knowledge, this is the first study that evaluated the uAm/Cr in dogs with leishmaniosis at the time of diagnosis and after anti-leishmanial treatment as a potential renal biomarker for monitoring treatment response and for assessing the severity of CanL. In the present study, the uAm/Cr was significantly reduced after antileishmanial treatment in dogs with leishmaniosis, regardless of the severity of the disease. Canine α-amylase is an enzyme with a molecular weight of 54 kilodaltons [35,36]. It has been hypothesized that amylase is filtrated by the glomerulus and partially reabsorbed by tubular epithelial cells with normally little or undetectable urinary amylase activity [36,37]. The strong positive correlation between the uAm/Cr and the UPC showed an association between the two variables. Thus, it can be hypothesized that a reduction in the uAm/Cr after anti-leishmanial treatment could indicate a recovery from kidney injury due to an improvement of glomerular and/or tubular damage. This possible mechanism should be additionally investigated in future studies. An interesting finding of the present study was that the decrease in uAm/Cr after leishmanicidal therapy in dogs with mild to moderate disease (Leishvet stages I and II) might suggest an improvement in renal damage not highlighted by the UPC in these stages. Another important aspect could be that the UPC can be influenced by active urinary sediment, but uAm/Cr probably is not, and therefore, it could be useful to measure it with or as an alternative to the UPC in this circumstance. Furthermore, this urinary marker needs to be evaluated in the medium and long-term follow-up to understand its utility as a marker for disease relapse or prognosis.

Antithrombin increased significantly post-treatment in dogs with mild to moderate disease and with severe to very severe disease. Laboratory determination of AT can be useful in the clinical evaluation and therapeutic management of patients with disseminated intravascular coagulation, nephrotic syndrome, and severe hepatopathy [38]. During CanL, a decreased concentration of AT can occur as in other protein losing nephropathy or during disseminated intravascular coagulation that has been reported in a leishmaniotic dog [39]. In the current study, the increase in AT could be secondary to the reduction in the proteinuria and/or to the improvement of the inflammatory state (since AT is also a negative acute phase protein) in the absence of liver failure and/or coagulation disorders. The data of this study suggest that an increase in this parameter can be expected as a response to leishmanicidal therapy.

The authors found that in dogs with severe leishmaniosis at the time of diagnosis TIBC was decreased and CRP was increased compared to reference interval and after treatment, TIBC increased and CRP decreased significantly. These results suggest an improvement in a pre-existing inflammatory condition in which CRP and TIBC could be increased and decreased, respectively. Previously, some authors have investigated the iron profile and its relationship with CRP in dogs infected with *L. infantum* at the time of diagnosis. These authors found that dogs with CanL had higher Ft and CRP and lower iron and TIBC compared to a group of healthy dogs and a group of dogs with diseases other than leishmaniosis. The conclusion of the study was that inflammation contributed in part to the iron status alterations found in CanL [25]. The results in the present study suggest that TIBC could be used as an inflammatory marker in severe disease to monitor an improvement in the inflammatory state following leishmanicidal treatment in alternative to CRP in laboratories that do not measure it.

PCR is a sensitive tool for the diagnosis of CanL, and one of the advantages of this technique is the variety of samples that can be analyzed [40,41,42,43]. Sensitivity and specificity vary according to the PCR techniques (conventional PCR, nested PCR, PCR-ELISA, and q-PCR), the target DNA sequence (highly repeated sequences, such as kinetoplast DNA minicircles or small subunit ribosomal RNA genes, as well as a variety of unique genes), and the type and number of different tissue evaluated [44,45] as well as the status of infection of the dogs (healthy versus sick) and the disease severity of the patient (Leishvet clinical staging) [26]. Bone marrow is one of the more sensitive tissues for the detection of *Leishmania* DNA [3], and assays based on PCR targeting kinetoplast DNA appeared to be the most sensitive technique for direct detection in infected tissues as previously described, and it was the technique used in this study [29,46]. Few studies have investigated the sensitivity and specificity of *Leishmania* q-PCR in different tissues, and only one study showed a sensitivity of 72.7% for *Leishmania* q-PCR in the bone marrow but no data available on specificity [40]. PCR assays with whole blood and urine samples are considered less sensitive than bone marrow [23,47,48,49]. Various studies have shown different sensitivity and specificity for *Leishmania* q-PCR in the blood, ranging from a sensitivity and a specificity of 11.3 and 96.6% to 61.5 and 100%, respectively [40,42]. There are no data available regarding the sensitivity and specificity for *Leishmania* q-PCR in the urine.

At diagnosis, the positive rate for the parasitic load in the blood and the urine was lower than in the bone marrow. This is not surprising because parasitic load is different in different tissues since *Leishmania* infection can be tissue-dependent in dogs, either by tissue tropism [2] or organ-specific immunity [50]. Of the samples tested, bone marrow had the highest parasite concentration, being one of the target organs for invasion and multiplication of parasites [51,52], while blood acts as a transport system rather than a reservoir organ [51,52]. Accordingly, blood is not the sample of choice for a molecular diagnosis of CanL [46].

After treatment, although the dogs in the study had a significant reduction in the parasitic load as shown by previous studies [19,53], more than half still had a positive bone marrow q-PCR, confirming that parasitological cure is difficult to achieve [54]. Interestingly, all dogs with a positive bone marrow q-PCR after leishmanicidal therapy, at the time of diagnosis, presented a positive *Leishmania* PCR in the blood or in the blood and urine. This study showed that a positive *Leishmania* q-PCR in the blood at diagnosis can be a predictor of a positive *Leishmania* q-PCR in the bone marrow after leishmanicidal therapy. The more probable reasons for these findings are likely to be related to more severe disease of these dogs and to a marked T helper 2-like response. The immune system plays a pivotal role in the *Leishmania* infection response to antileishmanial treatment [55]. In sick dogs, during long-term anti-*Leishmania* treatment, a clinical improvement is associated with a reduction in antibody level, a decrease in the parasite load, and an increase in interferon gamma as expression of a predominant cellular immune response over a humoral response [56,57]. Prior to appearance of clinical disease (clinical signs and/or clinicopathological abnormalities), a marked increase of *L. infantum* antibody levels (more than threefold elevation between monitoring samples) and of the parasitic load should be interpreted as a marker of disease progression or relapse, especially in dogs following discontinuation of treatment [3,26,56,58]. Based on the results of this study, at diagnosis, performing *Leishmania* q-PCR in the blood along with q-PCR in the bone marrow should be recommended as a useful monitoring tool to predict the parasitic load in the bone marrow at the end of treatment (which is the tissue with the highest parasitic density at the time of diagnosis). Therefore, these results are interesting for the clinical point of view because these dogs might be more susceptible, and they might be more at risk of disease relapse over time (regardless of the treatment protocol used) [44]. However, long-term studies should corroborate the present findings.

One-third of the dogs with leishmaniosis in the present study had a positive urine q-PCR for *Leishmania*. These findings confirmed the data from previous studies in which the presence of *L. infantum* DNA in the urine was reported in sick dogs naturally infected [23,59,60]; however, in the present study, the rate of positivity in the urine was lower [23,60]. Only dogs with advanced Leishvet stages III and IV had a positive urine q-PCR for *Leishmania,* suggesting that urine was not a good target tissue for diagnosing clinical leishmaniosis at its initial stages. The association between the *Leishmania* q-PCR in urine samples and Cr and SDMA suggests that a positive PCR in the urine is correlated with renal impairment and consequently an index of disease severity. These findings diverge from a previous study where no correlation between positive *Leishmania* PCR in the urine and presence of renal disease was found [59] but align with two other studies that highlighted a correlation between the occurrence of renal lesions and presence of measurable amounts of *Leishmania* DNA in the urine [23,60]. The discrepancy of results between the present study and the study by Franceschi and colleagues could be explained by the use of a different statistical test to calculate the correlation between *Leishmania* q-PCR in the urine and various renal markers. Based on the results of this study, *Leishmania* q-PCR in the urine, a simple and noninvasive test, should be recommended to identify dogs with severe disease at the time of diagnosis and to help in the management of sick dogs.

In the present study, WBC was significantly increased after treatment in dogs with severe to very severe leishmaniosis (Group 2) in contrast with a previous study in which the WBC did not change at the end of treatment [61]. The differences between the present results and those of Daza and colleagues could be due to the fact that a higher number of dogs included in the present study had more severe renal disease, different treatment protocol (MA + A or MT + A and not only with MA + A), and different WBC evaluation time (on day 30 or 60 and not on day 90). Some authors have described leukopenia in sick dogs with leishmaniosis [62,63]. Leukopenia may be due a multifactorial mechanism in which medullary dysfunction with diminished myelopoiesis, affected by an intense bone marrow parasitism as well as leukocyte recruitment and trapping into several organs and tissues are the major events [64]. It could also be related to secretion of suppressor cytokines triggered by *Leishmania* infection [65]. A human study on visceral leishmaniasis showed that, in the majority of sick patients, the WBC count was significantly lower at presentation and significantly increased post-treatment [66] In this human study, it is thought that the *Leishmania* parasite invades and multiplies in macrophages, potentially triggering an inflammatory response with the possible destruction of neutrophils, monocytes, and other types of leukocytes [67]. The results of this study showed that the evaluation of WBC in severe disease may not detect the presence of an inflammatory state; therefore, the combined evaluation of other markers of inflammation (such as APPs or TIBC) should be recommended.

The USG did not change significantly in dogs in Leishvet stages I and II; however, it significantly decreased after treatment in dogs with severe leishmaniosis. On the contrary, one study showed no change in USG in dogs with leishmaniosis treated with an antimonial (the majority of the dogs were not azotemic and had variable degree of proteinuria) [61]. Some authors have described a decrease in USG at the end of treatment with MT + A; however, the difference was not statistically significant [21]. A possible explanation for the reduction in USG could be an interference of anti-leishmanial drugs in the action of the antidiuretic hormone, as described in rats treated with MA, which developed disturbances in urine-concentrating capacity [68]. These results suggest that dogs with severe disease could have a decrease in USG as a consequence of anti-*Leishmania* therapy (both dogs treated with MA + A or with MT + A) and should be monitored in the following months to understand if the decrease in USG is reversible or irreversible and eventually if related to a specific therapeutic protocol used.

In the present study, Ft decreased significantly post-treatment in all the dogs with leishmaniosis. These results were similar to those of a study in which *L. infantum* infection in dogs was associated with a marked increase in serum Ft values, which returned to pre-infection values following treatment [16]. Another important aspect in this study was that the reduction in Ft was appreciated with both therapeutical protocols as previously described [18]. Ferritin is a good marker for monitoring the response to treatment as described by other authors [16,18], especially as shown by a study in which CRP and Ft concentrations within the reference intervals were usually associated with the absence of clinical signs and adequate response to treatment [69]. Despite this, Ft is not a good marker for establishing the severity of the disease.

At diagnosis, the best markers to establish the severity of CanL were the uAm/Cr, TIBC, AT, CRP, and *Leishmania* q-PCR in the urine. The uAm/Cr, TIBC, and AT have not previously been described as markers of disease severity. The role of CRP as marker of disease severity was demonstrated in a previous study with higher values in dogs with severe leishmaniosis [70]. Interestingly, PON-1, an APP indicative of oxidative damage associated with inflammation during leishmaniosis, was not one of the best markers for disease severity, even if in previous studies its decrease was related to the severity of the disease [71,72]. In the present study, the urine *Leishmania* q-PCR was identified as a marker of the severity of leishmaniosis and confirmed the results of previous studies and, therefore, its possible role in clinical practice as a screening test [23,60].

Since the low number of animals involved in the study (especially when the dogs were additionally divided into different groups according to the Leishvet clinical staging system) may be a limitation, larger studies would help to reduce the risk of type I error. However, a sample size and power analysis indicated that 30 subjects were sufficient to detect a statistically significant difference. Other limitations are represented by the short follow-up period, which does not allow conclusions to be drawn on more frequent relapses and/or a worse prognosis and the fact that the dogs enrolled in the study were classified in different LeishVet clinical stages and treated with two different protocols; therefore, a certain heterogenicity of the results obtained can be expected. Furthermore, it was possible to compare the two different treatment protocols only for dogs with severe to very severe disease. Future long-term studies are needed to investigate the prognostic value of these biomarkers in dogs with leishmaniosis.

## 5. Conclusions

The reduction of the uAm/Cr and the increase in AT between the time of diagnosis and post-leishmanicidal treatment are useful tools for monitoring the efficacy of the antileishmanial treatment at different clinical stages of CanL. Furthermore, uAm/Cr, TIBC, and AT were the best markers for identifying the severity of leishmaniosis at the time of diagnosis and should be further investigated as prognostic factors in long-term studies.

## Figures and Tables

**Table 1:** *Leishmania* parasitic load in different tissues and biomarkers expressed as median and interquartile range or mean and standard deviation at the time of diagnosis and post-treatment.

Variables	Diagnosis	Post-Treatment	*p*-Value	Effect Size
(Reference Interval)				(95% CI)
BM *Leishmania*-q-PCR (K copies/mL)				
(0–100)	57,600,000	15,560	<0.001	113 × 10^6^
	(27,560–[684 × 10^7^])	(0–[223 × 10^7^])		(431 × 10^5^; 499 × 10^6^)
Blood *Leishmania*-q- PCR (K copies/mL)				
(0–100)	40,100	0	<0.001	539,667
	(0–16,500,000)	(0–16,200)		(76,450; 1,945,050)
Urine *Leishmania*-q- PCR (K copies/mL)				
(0–100)	0	0	0.01	97,750
	(0–4,880,000)	(0–193,000)		(6780; 2,441,470)
WBC (cells/μL)				
(5410–12,590)	7535	9595	0.01	−1.83
	(2650–16,900)	(3000–25,230)		(−3.61; −0.36)
PON-1 (IU/L)				
(2.52–5.1)	3.42	4.01	0.02	−0.572
	(1.9–6.21)	(2.42–7.2)		(−1.048; −0.096)
Hp (mg/dL)				
(2–165)	163	110.5	0.11	39.967
	(1–533)	(1–617)		(−10.27; 90.21)
Ft (ng/mL)				
(80–272)	689	285	<0.001	470
	(183–4860)	(128–587)		(297.0; 701.5)
CRP (mg/dL)				
(0.001–0.4)	1.34	0.19	0.02	1.2422
	(0.01–7.56)	(0.01–13.73)		(0.209; 2.520)
TIBC (μL/dL)				
(336–424)	298.4 ± 80.4	317.8 ± 155	0.03	−19.47
				(−37.11; −1.82)
Iron (μL/dL)				
(95–213)	112.1 ± 58.7	116.6 ± 61.8	0.07	−4.57
				(−28.56; 19.43)
Alb (g/dL)				
(2.9–3.5)	2.55 ± 0.6	2.7 ± 0.5	0.15	−0.117
				(−0278; 0.044)
Glob (g/dL)				
(2.8–3.9)	4.4	3.6	<0.001	1.05
	(2.4–9.4)	(2.5–5.9)		(0.649; 1.749)
Fb (mg/dL)				
(184–313)	363 ± 152.4	323.8 ± 146.8	0.17	39.17
				(−17.29; 95.63)
AT (%)				
(103–138)	108.4 ± 26.5	126.3 ± 25.7	<0.002	−17.93
				(−27.86; −8.01)
Urea (mg/dL)				
(20–48)	31.5	28.5	0.38	3.00
	(14–331)	(19–224)		(−3.00; 19.00)
Cr (mg/dL)				
(0.7–1.4)	0.86	1	0.15	0.085
	(0.34–5.77)	(0.41–4.83)		(−0.015; 0.355)
SDMA (μL/dL)				
(0–15)	12.5	14	0.71	−0.499
				(−1.5; 2.0)
USG				
(1010–1051)	1035	1029	0.008	8.99
	(1014–1063)	(1004–1053)		(3.50; 13.00)
UPC				
(0.1–0.5)	0.7	0.4	0.02	0.90
	(0.1–27.7)	(0.1–16.20)		(0.15; 2.49)
FeNa (%)				
(0.1–1.0)	0.26	0.48	0.07	−0.155
	(0.002–3.43)	(0.04–4.74)		(−0.359; 0.029)
uAm/Cr				
(0.1–4.5)	98.5	5.6	<0.001	186.85
	(0.4–3700)	(0.4–2154)		(47.65; 660.45)
uG/Cr				
(2.0–8.5)	4.7	4.2	0.79	0.199
	(1.7–132.5)	(0.1–188.5)		(−1.349; 1.249)
uGGT/Cr				
(13–22)	49.7	40.05	0.02	12.45
	(3.5–214)	(3.8–122.8)		(2.25; 25.25)
uFerr/Cr				
(0–25)	27.5	20.5	0.93	0.499
	(0.1–429)	(0.1–300)		(−6.499; 13:00)

Alb, albumin; AT, antithrombin; BM, bone marrow; CI, confidence interval; CRP, C-reactive protein; Cr, creatinine; Ft, ferritin; Fg, fibrinogen; FeNa; fractional excretion of sodium; Glob, globulins; Hp, haptoglobin; K copies/mL, number of kinetoplast/mL; PON-1, paraoxonase-1; q-PCR, real-time-polymerase-chain-reaction; SDMA, symmetrical-dimethylarginine; TIBC, total iron-binding capacity; uAm/Cr, urinary amylase-to-creatinine ratio; uFerr/Cr, urinary ferritin-to-creatinine ratio; uG/Cr, urinary glucose-to-creatinine ratio; uGGT/Cr, urinary γ-glutamyl-transferase-to-creatinine ratio; UPC, urine protein-to-creatinine ratio; USG, urine specific gravity; WBC, white blood cell count.

**Table 2:** Inflammatory biomarkers and globulins in dogs with mild to moderate disease (Group 1) and dogs with severe to very severe disease (Group 2), which are expressed as median and interquartile range or mean and standard deviation at the time of diagnosis and post-treatment.

Variables	Group 1			Group 2			Difference at Diagnosis Between Groups
	Diagnosis	Post-tx	*p*-Value ES (95% CI)	Diagnosis	Post-tx	*p*-Value ES (95% CI)	*p*-Value ES (95% CI)
WBC							
(cells/μL)							
Median	7850	9190	0.30	6900	10,130	0.02	0.41
IQR	(300–16,900)	(5990–24,900)	−1.31	(2650–14,170)	(3000–5230)	−2.37	0.71
			(−4.19; 1.16)			(−4.98; −0.51)	(−2.299; 3.039)
PON-1							
(IU/L)							
Median	3.7	3.93	0.80	3.04	4.39	0.002	0.02
IQR	(2.2–6.2)	(2.9–6.3)	−0.07	(1.9–4.2)	(2.4–7.1)	−1.06	0.649
			(−0.84; 0.56)			(−1.86; −0.45)	(0.07; 1.34)
Hp							
(mg/dL)							
Median	172	115	0.92	138	91	0.08	0.90
IQR	(1–533)	(1–617)	−3.5	(1–341)	(1–269)	66.0	6.99
			(−72.0; 89.5)			(−11.9; 156.5)	(−91.9; 128.9)
Ft							
(ng/mL)							
Median	651	240	<0.001	813	303	<0.001	0.31
IQR	(183–2916)	(159–523)	401.5	(266–4860)	(128–579)	536.5	−170.9
			(176; 708)			(256.5; 1054.5	(−555.9; 182.0)
CRP							
(mg/dL)							
Median	0.24	0.01	0.28	3.78	0.22	0.044	0.006
IQR	(0.01–2.7)	(0.01–2.6)	0.38	(0.01–7.6)	(0.01–13.7)	2.75	−2.53
			(−0.56; 1.15)			(0.095; 4.77	(−4.79; −0.76)
TIBC							
(μL/dL)							
Mean	342.5	344.5	0.55	254.7	291.1	0.01	0.003
SD	±58.3	±51.9	−5.78	±76.7	±76.8	−34.5	83.99
			(−25.0; 26.0)			(−62.0; 12.5)	(25.00; 148.00)
Iron							
(μL/dL)							
Mean	132.1	133.9	0.80	92.1	99.3	0.41	0.05
SD	±60.6	±64.5	−2.75	±51.1	±55.7	−15.22	37.99
			(−40.5; 33.5)			(−45.5; 19.9)	(−1.99; 72.99)
Alb							
(g/dL)							
Mean	2.83	2.89	0.43	2.26	2.43	0.18	0.01
SD	±0.5	±0.5	−0.15	±0.6	±0.5	−0.19	0.59
			(−0.39; 0.29)			(−0.49; 0.05)	(0.10; 1.00)
Glob							
(g/dL)							
Mean	4.1	3.5	0.002	5.3	4.3	0.002	0.31
SD	±1.56	±0.66	0.70	±1.92	±0.76	1.40	−0.60
			(0.35; 1.75)			(0.60; 2.20)	(−2.29; 0.50)
Fb							
(mg/dL)							
Mean	299.9	289	0.64	426.1	358.7	0.18	0.04
SD	±100.5	±112.7	14	±171.7	±171.3	73.5	−110
			(−46; 73)			(−46.0; 184.9)	(−240.9; −1.00)
AT							
(%)							
Mean	122.5	137.7	0.007	94.2	114.9	0.03	0.003
SD	±19.2	±24.3	−13.9	±25.6	±22.4	−23	34.0
			(−26.0; −4.5)			(−40; −3)	(15.99; 47.99)

Alb, albumin; AT, antithrombin; CI, confidence interval; CRP, C-reactive protein; ES, effect size; Ft, ferritin; Fg, fibrinogen; Glob, globulins; Hp, haptoglobin; IQR, interquartile range; PON-1, paraoxonase-1; SD, standard deviation; TIBC, total iron-binding capacity; WBC, white blood cell count.

**Table 3:** *Leishmania* parasitic load in different tissues in dogs with mild to moderate disease (Group 1) and dogs with severe to very severe disease (Group 2) is expressed as median and interquartile range at the time of diagnosis and post-treatment.

Variables	Group 1			Group 2			Difference at Diagnosis Between Groups
	Diagnosis	Post-tx	*p*-Value ES (95% CI)	Diagnosis	Post-tx	*p*-Value ES (95% CI)	*p*-Value ES (95% CI)
*Leishmania*-q-PCR K copies/mL							
BM							
Median	3,920,000	0	<0.001	84,800,000	55,600	<0.001	0.65
IQR	(27,560–[684 × 10^7^])	(0–[223 × 10^7^])	757 × 10^5^	(34,000–[226 × 10^7^])	(0–[200 × 10^6^])	373 × 10^6^	−9,640,000
			(104 × 10^5^; 279.3 × 10^6^)			(148 × 10^5^; 867 × 10^6^)	(−651 × 10^6^; 392 × 10^5^)
Blood							
Median	0	0	0.02	111,000	0	0.003	0.03
IQR	(0–[159 × 10^4^])	(0–[162 × 10^3^])	68,276.4	(0–[165 × 10^5^])	(0–24,900)	651,990	−85,000
			(24,950; 1,110,500)			(98,000; 8,250,278)	(−100 × 10^4^; −398 × 10^3^)
Urine							
Median	0	0	1	2940	0	0.009	<0.001
IQR	0	(0–272)	−272	(0–[488 × 10^4^])	(0–[193 × 10^3^])	128,000	−2940
						(9180; 245 × 10^4^)	(−128 × 10^4^; −563 × 10^4^)

BM, bone marrow; CI, confidence interval; ES, effect size; IQR, interquartile range; K copies/mL, number of kinetoplast/mL; q-PCR, real-time-polymerase-chain-reaction.

**Table 4:** Renal biomarkers in dogs with mild to moderate disease (Group 1) and dogs with severe to very severe disease (Group 2) are expressed as median and interquartile range or mean and standard deviation at the time of diagnosis and post-treatment.

Variables	Group 1			Group 2			Difference at Diagnosis Between Groups
	Diagnosis	Post-tx	*p*-Value ES (95% CI)	Diagnosis	Post-tx	*p*-Value ES (95% CI)	*p*-Value ES (95% CI)
Urea							
(mg/dL)							
Median	26	27	0.91	45	40	0.29	0.02
IQR	(14–39)	(19–41)	0.49	(15–331)	(21–224)	16.6	−20.99
			(−3.9; 5.0)			(−7.5;51.9)	(−83.99; −3.00)
Cr							
(mg/dL)							
Median	0.83	0.94	0.93	1.540	1	0.09	0.13
IQR	(0.5–1.3)	(0.4–1.1)	−0.005	(0.3–5.8)	(0.6–4.8)	0.37	−0.59
			(−0.09; 0.11)			(−0.01; 0.79)	(−1200; 0.079)
SDMA							
(μl/dL)							
Median	12	11	0.421	151	7	0.81	0.02
IQR	(5–20)	(10–16)	−0.5	(9–51)	(11–44.1)	0.99	−4.0
			(−2.5; 1.5)			(−2.49; 4.25)	(−11.0; −0.99)
USG							
Median	1040	1038	0.26	1029	1017	0.008	0.15
IQR	(1014–1063)	(1019–1048)	6.0	(1015–1063)	(1004–1053)	11.49	9.0
			(−4.9; 11.9)			(5.49; 17.0)	(−2.99; 18.99)
UPC							
Median	0.4	0.3	0.67	2.5	0.8	0.01	<0.001
IQR	(0.1–1.0)	(0.1–2.0)	0.09	(0.3–27.7)	(0.1–16.2)	219	−2.1
			(−0.75; 0.49)			(0.5; 4.3)	(−7.09; −1.00)
FeNa							
(%)							
Median	0.28	0.5	0.25	0.25	0.47	0.18	0.88
IQR	(0.05–1.03)	(0.04–1.2)	−0.115	(0.02–3.4)	(0.1–4.7)	−0.235	0.02
			(−0.32; 0.07)			(−0.75; 0.19)	(−0.36; 0.19)
uAm/Cr							
Median	7.7	2.4	0.02	611.6	68.3	0.003	<0.001
IQR	(0.6–282.7)	(0.4–164.7)	5.0	(0.4–3700)	(2–2154.5)	604.77	−603.9
						(191.0; 937.5)	(−1482,2; −201.2)
uG/Cr							
Median	4.6	4.2	0.97	5.1	4	0.63	0.72
IQR	(2.8–11.5)	(3–48.5)	0.05	(1.7–132.5)	(0.1–188.5)	0.31	−0.19
			(−1.65; 1.15)			(−2.69; 3.15)	(−1.49; 1.59)
uGGT/Cr							
Median	41.2	38.2	0.29	72.1	41.9	0.15	0.11
IQR	(12.6–214)	(5.3–122.8)	12.9	(3.5–153.4)	(3.8–107.2)	14.2	−11.5
			(−7.5; 32.65)			(−7.2; 34.2)	(−46.9; 15.6)
uFerr/Cr							
Median	12	11	0.71	31	35	0.79	0.03
IQR	(0–321)	(0–40)	2.33	(2–429)	(1–300)	−2.5	−19.9
			(−3.49; 17.5)			(−21.0; 20.9)	(−33.9; −2.0)

CI, confidence interval; Cr, creatinine; ES, effect size; FeNa; fractional excretion of sodium; IQR, interquartile range; SD, standard deviation; SDMA, symmetrical-dimethylarginine; uAm/Cr, urinary amylase-to-creatinine ratio; uFerr/Cr, urinary ferritin-to-creatinine ratio; uG/Cr, urinary glucose-to-creatinine ratio; uGGT/Cr, urinary γ-glutamyl-transferase-to-creatinine ratio; UPC, urine protein-to-creatinine ratio; USG, urine specific gravity.

**Table 5:** Area under the curve, corresponding *p*-value, optimal Youden cutoff index with associated sensitivity and specificity.

Variables	AUC (95%CI)	*p*-Value	k	Se, Sp
uAm/Cr	0.87 (0.72–1.0)	<0.001	150.8	80%, 87%
TIBC	0.82 (0.67–0.98)	0.001	307.5	80%, 80%
AT	0.82 (0.66–0.99)	0.001	110	80%, 87%
Urine *Leishmania* q-PCR	0.80 (0.67–0.93)	<0.001	930	60%, 100%
CRP	0.80 (0.62–0.97)	0.003	1.5	73%, 80%
Alb	0.77 (0.59–0.94)	0.006	2.45	67%, 80%
Urea	0.76 (0.57–0.95)	0.008	42	53%, 100%
SDMA	0.76 (0.58–0.93)	0.008	13.5	67%, 73%
PON-1	0.75 (0.57–0.93)	0.009	3.48	67%, 80%
uFerr/Cr	0.74 (0.56–0.92)	0.01	20	80%, 67%
Blood *Leishmania* q-PCR	0.73 (0.55–0.91)	0.01	57,600	67%, 80%
Fb	0.72 (0.55–0.91)	0.02	350	67%, 73%
Glob	0.61 (0.40–0.82)	0.15	6	47%, 87%

UAm/Cr, urinary amylase-to-creatinine ratio; TIBC, total iron-binding capacity; AT, antithrombin; q-PCR, real-time-polymerase-chain-reaction; CRP, C-reactive protein; Alb, albumin; SDMA, symmetrical-dimethylarginine; PON-1, paraoxonase-1; uFerr/Cr, urinary ferritin-to creatinine ratio; Fb, fibrinogen; Glob, globulins; AUC, area under the curve; CI, confidence interval; k, cutoff value; Se, sensitivity; Sp, specificity.

## Data Availability

The complete dataset is available upon reasonable request.

## Supplementary Materials

The following supporting information can be downloaded at: https://www.mdpi.com/article/10.3390/vetsci11110517/s1, Table S1: Individual characteristics and treatment protocol for each dog with leishmaniosis at diagnosis; Table S2: Individual characteristics for each dog with leishmaniosis at diagnosis and post leishmanicidal treatment (at day 30 if treated with meglumine antimoniate and allopurinol or at day 60 if treated with miltefosine and allopurinol); Table S3: *Leishmania* parasitic load in different tissues, inflammatory and renal biomarkers in dogs with severe to very severe disease treated with MA + A (Group 3) or with MT + A (Group 4) expressed as median and interquartile range or mean and standard deviation at the time of diagnosis and post-treatment.
