# Supplementary material for: Short Term Treatment Monitoring of Renal and Inflammatory Biomarkers with Naturally Occurring Leishmaniosis: A Cohort Study of 30 Dogs
Source: Vet Sci. 2024 Oct 22;11(11):517. doi: 10.3390/vetsci11110517 (PMC11598865; doi:10.3390/vetsci11110517)
Supplement: Supplementary file 1 [file vetsci-11-00517-s001.zip › vetsci-3205188-supplementary.pdf]

Table S1. Individual characteristics and treatment protocol for each dog with leishmaniosis at diagnosis.

Dog ID	LeishVet Clinical stage	IRIS stage	ELISA positivity (%)	Bone marrow q-PCR (K copies/ml)	Blood q-PCR (K copies/ml)	Urine q-PCR (K copies/ml)	Tx
**1**	I	I, NPR, NH	38.7	140800000	0	0	MA+A
**2**	I	I, NPR, PreH	36.7	150800000	0	0	MA+A
**3**	IIa	I, BPR, NH	41.8	15880000	0	0	MA+A
**4**	IIa	I, BPR, PreH	50.8	39200000	33500	0	MA+A
**5**	IIa	I, BPR, PreH	15.6	27500	0	0	MA+A
**6**	IIa	I, BPR, PreH	16.7	132800	0	0	MA +A
**7**	IIa	I, BPR, PreH	37.0	5000000	0	0	MA+A
**8**	IIa	I, BPR, H	23.9	10120000	46700	0	MT+A
**9**	IIa	I, BPR, H	43.1	6840000000	1590000	0	MA+A
**10**	IIb	I, BPR, PreH	34.4	7600000	0	0	MA+A
**11**	IIb	I, BPR, H	26.0	76000000	47300	0	MA+A
**12**	IIb	I, BPR, H	56.3	198000000	9720000	0	MA+A
**13**	IIb	I, PR, NH	40.4	3372000	0	0	MA+A
**14**	IIb	I, PR, NH	46.6	556000000	793000	0	MA+A
**15**	IIb	I, PR, PreH	47.8	90000000	81400	0	MA+A
**16**	III	I, PR, NH	45.6	25280000	1000000	0	MA+A
**17**	III	I, PR, PreH	42.8	2256000000	1200000	2940	MA+A
**18**	III	I, PR, PreH	43.5	125400	556	0	MA+A
**19**	III	I, PR, H	52.7	740000000	298000	1860	MA+A
**20**	III	I, PR, H	22.9	304000	0	0	MA+A
**21**	III	I, PR, H	44.5	1564000000	16500000	4888000	MT+A
**22**	III	II, BPR, H	37.8	2356000	10200	11700	MA+A
**23**	III	II, PR, PreH	12.1	37400	0	16500	MA+A
**24**	IV	I, PR, SH	11.7	34000	0	0	MA+A
**25**	IV	II, PR, NH	53.1	996000000	15000000	179000	MA+A
**26**	IV	II, PR, H	20.9	14640000	67900	0	MT+A
**27**	IV	II, PR, SH	35.0	322400000	85000	0	MT+A
**28**	IV	III, PR, PreH	28.6	84800000	111000	128000	MT+A
**29**	IV	IV, PR, SH	46.3	1716000000	3830000	1140000	MT+A
**30**	IV	IV, PR, SH	34.2	192000000	303000	192000	MT+A

ID, identication number; NPR, non-proteinuric (< 0.2); BPR, border-proteinuric (0.2 - 0.5); PR, proteinuric (> 0.5); NH, normotensive (< 140 mmHg); PreH, pre-hypertensive (140 - 159 mmHg); H, hypertensive (160 - 179 mmHg); SH, severely hypertensive (≥ 180 mmHg); ELISA test, *Leishmania* ELISA test; q-PCR, real-time-polymerase-chain-reaction; K copies, number of kinetoplast copies; Tx, treatment protocol; MA+A, meglumine antimoniate+alloprinol; MT+A, miltefosine+allopurinol.

Table S2. Individual characteristics for each dog with leishmaniosis at diagnosis and post leishmanicidal treatment (at day 30 if treated with meglumine antimoniate and allopurinol or at day 60 if treated with miltefosine and allopurinol).

**Dog ID Tx**	**LeishVet Clinical stage**		**IRIS stage**		**Bone marrow q-PCR (K copies/ml)**		**Blood q-PCR (K copies/ml)**		**Urine q-PCR (K copies/ml)**	
	**Dx**	**Post-Tx**	**DX**	**Post- Tx**	**Dx**	**Post- Tx**	**Dx**	**Post-Tx**	**Dx**	**Post-Tx**
**1** MA+A	I	I	I, NPR, NH	I, NPR, NH	140800000	0	0	0	0	0
**2** MA+A	I	I	I, NPR, PreH	I, NPR, NH	150800000	0	0	0	0	0
**3** MA+A	IIa	IIa	I, BPR, NH	I, BPR, NH	15880000	0	0	0	0	0
**4** MA+A	IIa	IIa	I, BPR, PreH	I, BPR, PreH	39200000	5360	33500	0	0	0
**5** MA+A	IIa	IIa	I, BPR, PreH	I, BPR, NH	27500	0	0	0	0	0
**6** MA+A	IIa	I	I, BPR, PreH	I, NPR, NH	132800	0	0	0	0	0
**7** MA+A	IIa	IIa	I, BPR, PreH	I, BPR, PreH	5000000	0	0	0	0	0
**8** MT+A	IIa	IIa	I, BPR, H	I, BPR, PreH	10120000	664000	46700	0	0	0
**9** MA+A	IIa	III	I, BPR, H	I, PR, H	6840000000	2224000000	1590000	162000	0	272
**10** MA+A	IIb	IIb	I, BPR, PreH	I, BPR, PreH	7600000	0	0	0	0	0
**11** MA+A	IIb	IIb	I, BPR, H	I, BPR, H	76000000	34640	47300	0	0	0
**12** MA+A	IIb	IIb	I, BPR, H	I, BPR, PrH	198000000	8530000	9720000	0	0	0
**13** MA+A	IIb	IIa	I, PR, NH	I, PR, NH	3372000	44400	0	0	0	0
**14** MA+A	IIb	IIa	I, BPR, NH	I, BPR, NH	556000000	784000	793000	0	0	0
**15** MA+A	IIb	IIa	I, PR, PreH	I, BPR, NH	90000000	0	81400	0	0	0
**16** MA+A	III	I	I, PR, NH	I, NPR, PreH	25280000	0	1000000	0	0	0
**17** MA+A	III	IIb	I, PR, PreH	I, PR, PreH	2256000000	0	1200000	0	2940	0
**18** MA+A	III	I	I, PR, PreH	I, NPR, PreH	125400	3768	556	0	0	0
**19** MA+A	III	IIb	I, PR, H	I, PR, H	740000000	2424000	298000	0	1860	0
**20** MA+A	III	III	I, PR, H	II, NPR/PreH	304000	0	0	0	0	0
**21** MT+A	III	IIb	I, PR, H	I, BPR, PreH	1564000000	55600	16500000	0	4888000	0
**22** MA+A	III	IIa	II, BPR, H	I, BPR, PreH	2356000	25760	10200	0	11700	0
**23** MA+A	III	III	II, PR, PreH	II, BPR, PreH	37400	0	0	0	16500	0
**24** MA+A	IV	III	I, PR, SH	I, PR, H	34000	0	0	0	0	0
**25** MA+A	IV	IV	II, PR, NH	I, PR, NH	996000000	223200	15000000	0	179000	0
**26**	IV	III	II, PR, H	I, PR, H	14640000	464000	67900	0	0	0

MT+A										
**27** MT+A	IV	III	II, PR, SH	I, PR, SH	322400000	22040000	85000	0	0	0
**28** MT+A	IV	IV	III, PR, PreH	II, PR, PreH	84800000	878000	111000	0	128000	0
**29** MT+A	IV	IV	IV, PR, SH	III, PR, SH	1716000000	20040000	3830000	24900	1140000	193000
**30** MT+A	IV	IV	IV, PR, SH	III, PR, H	192000000	22600000	303000	0	192000	0

ID, identification number; Tx, treatment protocol; Dx, diagnsosi; Post-Tx;, post-treatment; MA+A, meglumine antimoniate+alloprinol; MT+A, miltefosine+allopurinol; NPR, non-proteinuric (< 0.2); BPR, border-proteinuric (0.2 - 0.5); PR, proteinuric (> 0.5); NH, normotensive (< 140 mmHg); PreH, pre-hypertensive (140 - 159 mmHg); H, hypertensive (160 - 179 mmHg); SH, severely hypertensive (≥ 180 mmHg); real-time-polymerase-chain-reaction; K copies, number of kinetoplast copies.

Table S3. *Leishmania* parasitic load in different tissues, inflammatory and renal biomarkers in dogs with severe to very severe disease treated with MA + A (Group 3) or with MT + A (Group 4) expressed as median and interquartile range or mean and standard deviation at the time of diagnosis and post-treatment.

**Variables**	**Group 3 (n=9)**			**Group 4 (n=6)**		
	Diagnosis	Post-tx	*P-value*	Diagnosis	Post-tx	*P-value*
***Leishmania*-q-PCR** K copies/mL						
BM						
Median	2356000	0	0.004	257200000	11458000	0.0313
IQR	739875600	25760		1142000000	21563000	
Urine						
Median	1860	0	0.059	160000	0	0.1003
IQR	11700	0		871000	0	
Blood						
Median	10200	0	0.0360	207000	0	0.0312
IQR	1000000	0		2856750	0	
**WBC** (cells//µl)						
Median	6.9	10.57	0.0039	8.36	9.09	0.8438
IQR	3.83	3.72		4.58	3.49	
**PON-1** (IU/L)						
Median	3.03	4.39	0.0039	3.43	4.25	0.1056
IQR	0.81	1.22		0.76	0.76	
**Hp** (mg/dL)						
Median	267	118	0.0976	102	37.5	0.7874
IQR	228	109		43.5	154.5	
**Ft** (ng/ml)						
Median	619	303	0.0391	1017.5	290	0.0313
IQR	537	102		557.5	60.5	
**CRP** (mg/dL)						
Median	2.5	0.29	0.5286	5.08	0.09	0.0312
IQR	4.74	1.08		3.91	0.36	
**TIBC** (µl/dL)						
Mean	281	322	0.0111	215.2	244.8	0.2149
SD	63.3	62.1		83.3	77.9	
**Iron** (µl/dL)						
Mean	105	101.1	0.8901	72.7	96.7	0.1091
SD	58.4	65.8		33.4	41.7	
**Alb** (g/dL)						
Mean	2.3	2.5	0.1631	2.18	2.33	0.5177
SD	0.62	0.44		0.59	0.51	
**Glob**						

(g/dL)						
Mean	5.9	4.4	0.011	4.9	3.7	0.0689
SD	2.06	0.75		1.69	0.64	
**Fb**						
(mg/dL)						
Mean	366.3	357.2	0.8093	515.7	361	0.192
SD	153.2	170.3		170.1	189.1	
**AT**						
(%)						
Mean	102.2	121.3	0.1812	82.2	105-2	0.08
SD	30.4	21.9		7.8	21.1	
**Urea**						
(mg/dL)						
Median	37	39	0.999	151.5	132.3	0.1563
IQR	19	20		103.5		
**Cr**						
(mg/dL)	0.82	0.79	0.999	2.89	1.74	0.035
Median	0.93	0.42		2.68	2.74	
IQR						
**SDMA**						
(µl/dL)						
Median	14	17	0.153	27.6	20	0.1563
IQR	4	4		27.9	22	
**USG**						
Median	1029	1015	0.009	1026	1019.5	0.5625
IQR	18	6		11.7	7.5	
**UPC**						
Median	1.5	0.4	0.0117	8.5	3.9	0.3125
IQR	3.7	0.7		9.1	7.6	
**FeNa**						
(%)						
Median	0.17	0.47	0.6523	0.48	1.09	0.3125
IQR	0.51	0.27		0.52	1.68	
**uAm/Cr**						
Median	379.1	27.8	0.0078	1345.2	214.8	0.2188
IQR	1414.3	149.5		870.3	890.3	
**uG/Cr**						
Median	5.1	3.8	0.0969	4.35	5.9	0.1563
IQR	2.5	2.8		2.8	6.2	
**uGGT/Cr**						
Median	43.5	41.9	0.3008	81.4	43.4	00625
IQR	46.5	37.3		13.1	18.8	
**uFerr/Cr**						
Median	30	46	0.9055	33.5	34.5	0.8438
IQR	29	37		20	22	

Alb, albumin; A, allopurinol; AT, antithrombin; BM, bone marrow; CI, confidence interval; CRP, C-reactive protein; Cr, creatinine; Ft, ferritin; Fg, fibrinogen; FeNa; fractional excretion of sodium; Glob, globulins; Hp, haptoglobin;; K copies/ml, number of kinetoplast/ml; MA, meglumine antimoniate; MT, miltefosine; PON-1, paraoxonase-1; q-PCR, real-time-polymerase-chain-reaction; SDMA, symmetrical-dimethylarginine; TIBC, total iron-binding capacity; uAm/Cr, urinary amylase-to-creatinine ratio; uFerr/Cr, urinary ferritin-to-creatinine ratio; uG/Cr, urinary glucose-to-creatinine ratio; uGGT/Cr, urinary γ-glutamyl-transferase-to-creatinine ratio; UPC, urine protein to creatinine ratio; USG, urine specific gravity; WBC, white blood cell count.
